# Diagnostic value of T2 relaxation time for hepatic iron grading in rat model of fatty and fibrotic liver

**DOI:** 10.1371/journal.pone.0278574

**Published:** 2022-12-05

**Authors:** Mingli Jin, Yin Jiang, Qi Zhao, Zhihua Pan, Fang Xiao

**Affiliations:** 1 Department of Radiology, The Second Affiliated Hospital of Chengdu Medical College, China National Nuclear Corporation 416 Hospital, Cheng du, Sichuan, People’s Republic of China; 2 Department of Radiology, The First Affiliated Hospital of USTC, Division of Life Sciences and Medicine, University of Science and Technology of China, Hefei, Anhui, People’s Republic of China; Cornell University, UNITED STATES

## Abstract

The objective of this study was to assess the quantitative diagnostic value of T2 relaxation time for determining liver iron grades in the presence of fat and fibrosis. Sixty Sprague-Dawley (SD) male rats were randomly divided into control (10 rats) and model (50 rats) groups. The model group of coexisting iron, steatosis, and liver fibrosis was induced by intraperitoneal injection of carbon tetrachloride (CCl_4_) dissolved in edible vegetable oil (40% v/v). The control group received an intraperitoneal injection of 0.9% saline. All rats underwent multi-echo gradient and spin echo (M-GRASE) magnetic resonance imaging, and the T2 relaxation time of the liver was measured. The rats were killed immediately after imaging, and liver specimens were extracted for histological evaluation of steatosis, iron, and fibrosis. The relationship and differences between T2 relaxation time and liver fibrosis stage, as well as the pathological grade of hepatic steatosis, were assessed by Spearman’s rank correlation coefficient, non-parametric Mann–Whitney test, and the Kruskal–Wallis test. The area under the receiver operating characteristic curve and interaction analysis were used to quantify the diagnostic performance of T2 relaxation time for detecting different degrees of liver iron grades. Six normal control rats and 34 model rats were included in this study. Fibrosis stages were F0 (n = 6), F1 (n = 6), F2 (n = 8), F3 (n = 10), and F4 (n = 10). Steatosis grades were S0 (n = 5), S1 (n = 8), S2 (n = 12), and S3 (n = 15). Hepatocyte or Kupffer cell iron grades were 0 (n = 7), 1 (n = 9), 2 (n = 12), 3 (n = 10), and 4 (n = 2). The liver fibrosis stages were positively correlated with the iron grades (*P < 0*.*01*), and the iron grades and fibrosis stages were negatively correlated with the T2 relaxation time (*P < 0*.*01*). The T2 relaxation times exhibited strongly significant differences among rats with different histologically determined iron grades (*P < 0*.*01*). Pairwise comparisons between each grade of liver iron indicated significant differences between all iron grades, except between grades 0 and 1, and between grades 1 and 2 (*P > 0*.*05*). The T2 relaxation time of the liver had an area under the receiving operating characteristic curve (AUC) of 0.965 (95% CI 0.908–0.100, *P < 0*.*001*) for distinguishing rats with a pathological grade of hepatic iron (grade ≥ 1) from those without, an AUC of 0.871 (95% CI 0.757–0.985, *P < 0*.*001*) for distinguishing rats with no iron overload (grade ≤ 1) from rats with moderate or severe iron overload (grade ≥ 2), and an AUC of 0.939 (95% CI 0.865–1.000, *P < 0*.*001*) for distinguishing rats with no to moderate iron overload (grade ≤ 2) from rats with severe iron overload (grade 3). The interaction of different pathological grades of iron, steatosis, and fibrosis has a negligible influence on the T2 relaxation time (*P > 0*.*05*). In conclusion, T2 relaxation time can assess histologically determined liver iron grades, regardless of coexisting liver steatosis or fibrosis; therefore, it is suitable for distinguishing between the presence and absence of iron deposition and it is more accurate for higher iron grading.

## Introduction

Hepatic fibrosis refers to a common pathological stage in acute and chronic hepatic injury characterized by hepatic excess accumulation of extracellular matrix (ECM) components, which will develop into cirrhosis and liver failure, sometimes progressing to hepatocellular carcinoma (HCC) [1–3]. The typical histological features of liver fibrosis mainly include hepatocellular necrosis, inflammation, and iron deposition [4, 5]. Iron-loading can accelerate the Fenton reaction, increase the generation of reactive oxygen species (ROS), and induce fibrosis-promoting signals in the parenchymal and non-parenchymal cells, all of which will severely damage cells and tissues and accelerate the progression of liver fibrosis to cirrhosis [5–7]. Therefore, it is very important and useful to detect and quantify liver iron content.

At present, liver biopsy is the gold standard for liver iron content quantification, but it carries a risk of sampling error or infection [8, 9]. Serum iron (SI), transferrin saturation (SF), and serum ferritin levels are indicators of total body iron stores, but they are unsuitable for use in cases with inflammation, hepatic injury, and vitamin C deficiency, and they cannot directly determine hepatic iron stores [8–11]. Magnetic resonance imaging (MRI) has the potential to aid in the diagnosis and quantification of liver iron. Many previous clinical studies have used MRI to quantitatively evaluate the brain, spleen, liver, and cardiac iron content [12, 13]. One common technique is T2 relaxometry, which is based on the measurement and imaging of proton transverse relaxation time (T2 or R2) within the liver [14]. Chronic liver disease (which can be caused by viral hepatitis [hepatitis B and C infections], alcoholic liver disease [ALD], non-alcoholic fatty liver disease [NAFLD], and non-alcoholic steatohepatitis [NASH]), iron overload (mainly mildly and moderately elevated hepatic iron concentration), steatosis, and fibrosis can theoretically result in reduced T2 relaxation time [10, 15, 16]. T2-MRI may aid in the diagnosis and quantification of liver iron.

However, confounders must be considered. Two studies found that liver iron content measured by T2-MRI is unaffected by the stage of liver fibrosis and necroinflammation [17, 18], whereas the results of another study revealed that T2-MRI measurements appear to be affected by different degrees of hepatic fibrosis [19]. Several studies have indicated that the coexistence of steatosis and iron in the liver can potentially confound T2 measurements [20, 21]. The results of previous studies may indicate that T2 relaxation time is a nonspecific surrogate marker for liver iron content, with liver fat and liver fibrosis as possible confounders. Therefore, the purpose of our study was to use a liver fibrosis rat model induced by carbon tetrachloride (CCl_4_) to investigate the presence and distribution of iron and steatosis in liver fibrosis, investigate the value of T2 relaxation time for grading liver iron in the presence of liver steatosis and fibrosis, and study the effect of hepatic fibrosis and steatosis on the distinction of liver iron grades.

## Materials and methods

### Materials

#### Carbon tetrachloride edible blended oil suspension (solution)

CCl_4_ solution (99.5% concentration, 80.4 ml) was diluted in edible blended oil (200 ml) in a brown bottle to obtain a suspension concentration of 40%. CCl_4_ was purchased from Jiangsu Qiangsheng Functional Chemical Co, Ltd. (Because high-purity CCl_4_ solution has strong volatility and toxicity, experimenters wore plastic gloves and disposable masks throughout the process and kept the laboratory ventilated). The suspension was placed in a cool, dry environment away from light before MRI measurement.

The laboratory animal production licenses were SCXK (Chuan) 2013–17, SCXK (Chuan) 2013–181, and SCXK (Chuan) 2013–065. Sixty male Sprague-Dawley (SD) rats (2–3 months old), weighing 202 ± 3.2 g, were provided by the Experimental Animal Centre of Southwest Medical University (Sichuan, China). The experimental procedures mentioned in this study were approved by the Ethical Committee for the Experimental Use of Animals at Southwest Medical University (Sichuan, China).

MRI was performed with an 8-channel wrist coil on a 3.0 Tesla MRI scanner (Achieva 3.0T, Philips Healthcare, The Netherlands). The pulse sequence was M-GRASE, and the parameters were: TE = 17 ms × 4 echos (TE1 = 17, TE2 = 34, TE3 = 51, and TE4 = 68 ms), TR = 1552 ms, slice thickness = 2.5 mm, slice gap = 1 mm, matrix size = 240 × 240, field of view (FOV) = 100 × 60 mm, number of excitations (NEX) = 1, and the scanning ranged from the top of the diaphragm to the lower edge of the kidneys.

### Methods

#### Animal model and MRI

All rats were fed a normal diet and housed under controlled conditions. Rats were injected with the 40% CCl_4_ edible mixed oil suspension (0.3 ml/100 g) subcutaneously into the abdominal cavity or the inside of the hind legs twice a week. From 6 to 12 weeks of modeling, 1–6 rats with light weight, darker coat color, and listlessness were selected for MRI scan every week. For MRI, the rats were anesthetized by subcutaneous injection of 2% sodium pentobarbital (0.2 ml/100 g). The rats were sacrificed via subcutaneous injection of 4% sodium pentobarbital (0.2 mL/100 g) after scanning.

#### Histopathological assessment

The rats were killed immediately after MRI, and liver specimens were extracted, routinely processed with normal saline solution flushing and 10% buffered neutral formalin fixation and embedded in paraffin. Sections of the liver tissue were stained with hematoxylin and eosin (H&E) for histological evaluation of steatosis, Perl’s iron stain (Prussian blue reaction) for evaluation of iron grade, and Masson’s trichrome stain for evaluation of fibrosis. The outcomes of H&E, Prussian blue, and Masson’s trichome staining were independently evaluated by two experienced pathologists who were blinded to each other’s findings and MRI data. The histopathological outcome of each specimen represents the consensus opinion.

Diagnostic criteria for pathological grading of hepatic steatosis [22] were as follows: hepatic fat accumulation was evaluated using low-to-medium-power magnification estimation of hepatocyte involvement by macrosteatosis or microsteatosis with grade 0 at <5% of parenchyma, grade 1 at 5%–33%, grade 2 at 34%–66%, and grade 3 at >66%. Hepatocellular iron was graded as follows [23]: grade 0 if there are no iron particles; grade 1 if iron particles can be seen in a few hepatocytes; grade 2 if iron particles can be seen in 5%–10% of hepatocytes; grade 3 if >40% iron particles can be seen in hepatocytes; and grade 4 if abundant iron particles can be seen in most hepatocytes. Liver fibrosis was assessed by Scheuer Classification [24]: F0 means no fibrosis; F1 means fibrous expansion of portal areas without short fibrous septa; F2 means fibrous expansion of most portal areas with occasional portal to portal bridging; F3 means marked bridging with occasional nodules and incomplete cirrhosis; and F4 means cirrhosis.

#### Quantitative image analysis

**Fig 1:**
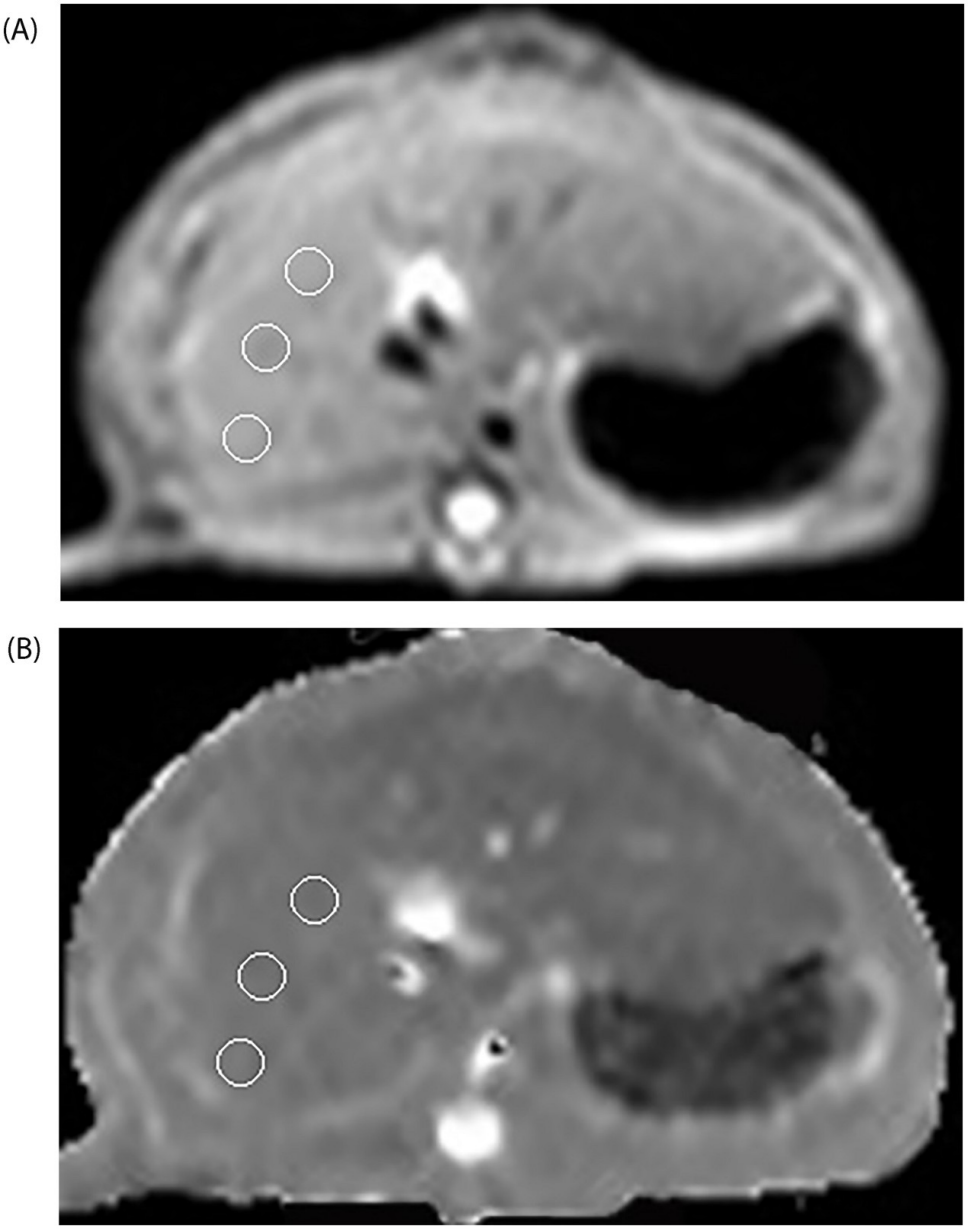
The position of a region of interest (ROI) on the T2 map of liver. Representative axial multi-echo gradient and spin echo (TE = 17 ms) T2 MRI imaging (a) and T2 map (b) of one SD rat illustrating the position of ROIs in the liver. Each ROI is located at the center of the largest cross-section of the liver and avoids artifacts, bile ducts, large blood vessels, and focal liver damage. Each ROI is first placed on the TE = 17 ms image and then copied to the same area of the T2 map. Three ROIs are placed in different positions on the same axial image.

Raw image data were manually transferred to a PC workstation (Philips Extended MR WorkSpace, release 2.6.3.4; Philips Medical Systems), and the system automatically constructed a T2 map. Because of the inferiority in soft tissue contrast of T2 mapping, a circular region of interest (ROI) of 10 mm^2^ was placed on the T2 MRI (TE: 17 ms) first, and then the ROI of the same area was copied to the T2 map. The ROI was selected to be located in the larger cross-section of the liver and at the center of the images, and it was also selected to avoid artifacts, bile ducts, large blood vessels, and focal liver damage to reduce error. The ROI was set at three different locations of the same cross-section image of the T2 map. The position of the ROI is shown in Fig 1A and 1B. The T2 relaxation times of the MR images were independently measured by the two experienced radiologists, who were blinded to each other’s MR assessments and the results of the histological examination. The mean of the measurements was taken as the final value.

#### Statistical analysis

The statistical analysis was performed by IBM SPSS 22.0 package program and GraphPad Prism 7. Count data were presented as frequency and percentage, and non-normally distributed data were summarized as median, maximum, and minimum. The degree of correlation between non-normally distributed data or ordinal variables was assessed by Spearman’s rank correlation coefficient and Kendall’s tau-b. The difference between the pathological hepatic iron grade and those determined by the T2 relaxation time was compared by applying the non-parametric all pairwise test and the Kruskal–Wallis test. The receiver operating characteristic (ROC) analysis assessed the diagnostic accuracy. The diagnostic accuracy of the T2 relaxation time for the assessment of pathological hepatic iron was evaluated by the area under the receiver operating characteristic (AUROC) curve. The main and interaction effects between steatosis, fibrosis, and iron on the T2 relaxation time were assessed by factorial analysis of variance design. Only two rats had grade 4 pathological hepatic iron; so, the grade 4 rats were consolidated with the grade 3 rats statistically due to the small sample numbers. A p-value less than 0.05 was defined as statistically significant for all tests.

## Results

### Animal models and pathological results

**Fig 2:**
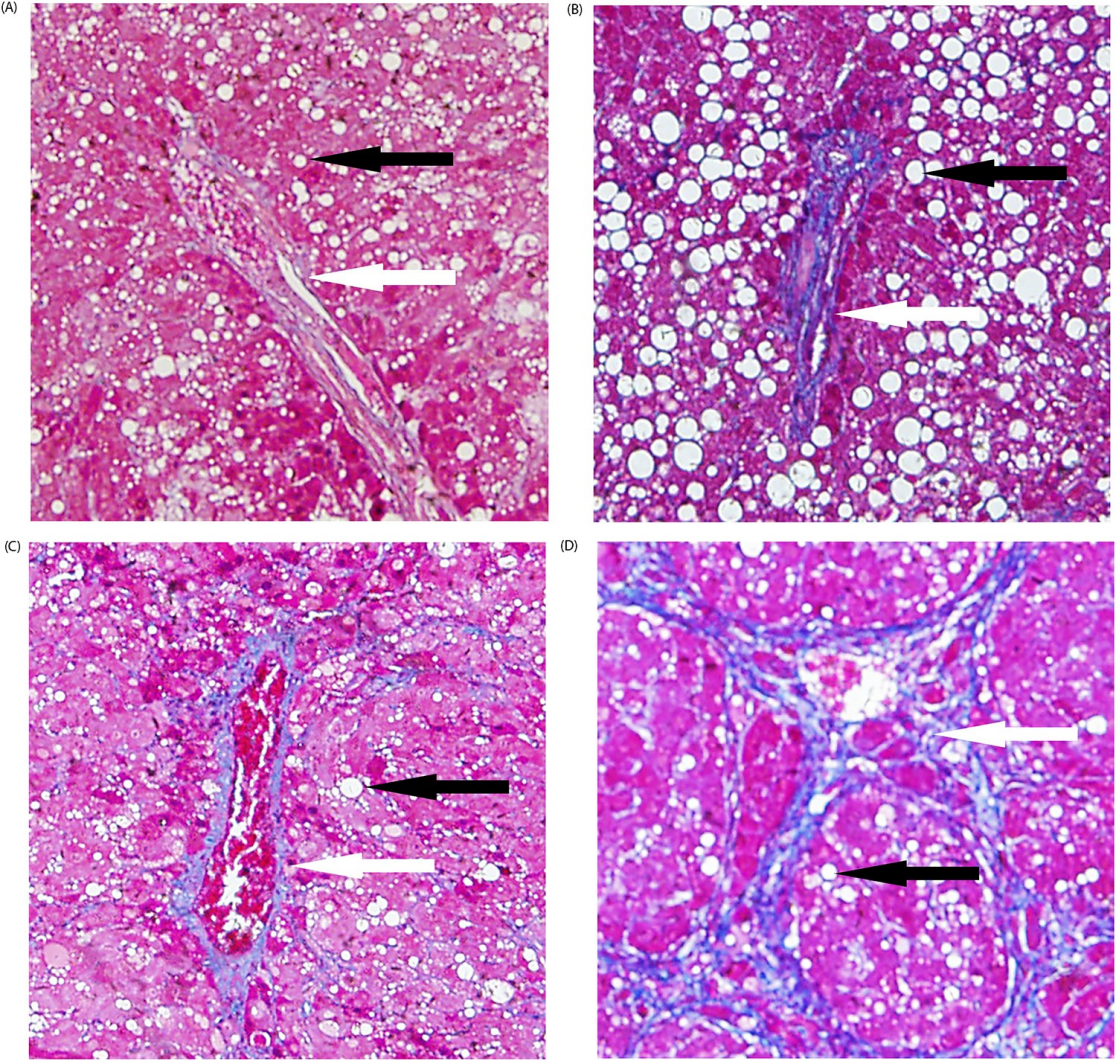
Liver fibrosis in one SD rat, where (A)–(D) correspond to stages F1–F4 (Masson stain, original magnification, 200 x). Blue staining (white arrow) shows collagen fibers, and the black arrow indicates a fat vacuole. As the stage of liver fibrosis progresses, the accumulation of blue-stained collagen fibers gradually increases, which is associated with different degrees of steatosis.

**Fig 3:**
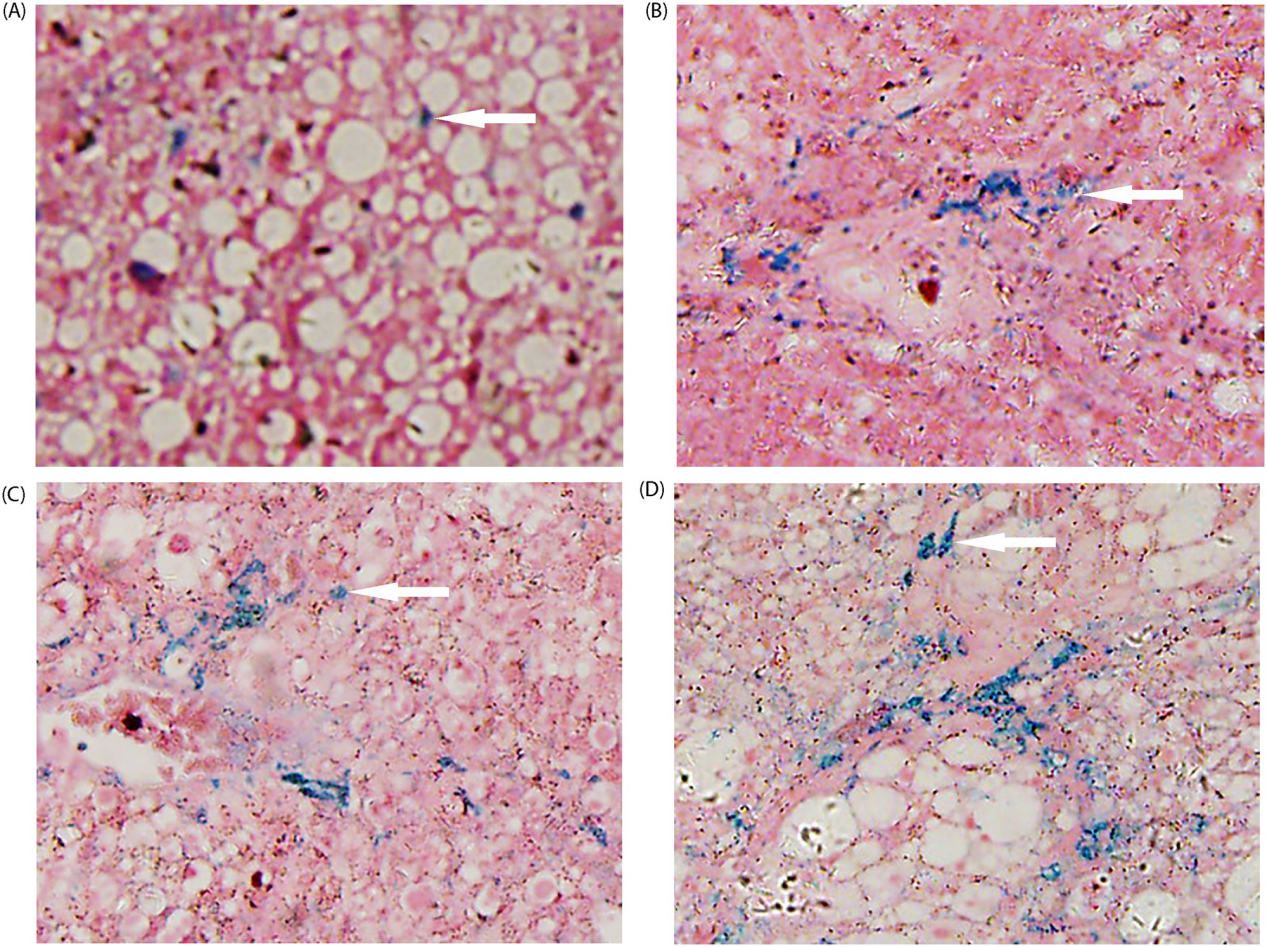
Perl’s iron stain for rats with different stages of liver fibrosis, where (A)–(D) correspond to stages 1–4 (original magnification, 200 x). The histology stain indicates that the ratio of hemosiderin (blue stain, white arrow) increases gradually as the liver fibrosis stage advances.

Twenty of the 60 rats died as a result of acute liver failure, infection, and other causes following CCl_4_ injection, with a mortality rate of 33.3%. Forty rats underwent pathological evaluation and MRI, and the results are presented in Table 1. Figs 2A–2D and 3A–3D show the pathological grading of liver iron and the stage of liver fibrosis. The liver fibrosis stage was positively correlated with iron grade (*r = 0*.*810*, *P < 0*.*05*) and not correlated with steatosis *(P > 0*.*05)*. There were also weak correlations between steatosis and liver iron (*r = 0*.*356*, *P < 0*.*05*), and there were significant increases in the pathological grade of hepatic iron and steatosis along with the increase of fibrosis staging.

**Table 1:** Research data for the 40 study rats.

	N (%)	T2 relaxation time (ms), Median (maximum, minimum)
Steatosis grade (H&E)		
0	5 (13%)	59.10 (60.70, 56.60)
1	8 (20%)	56.45 (61.30, 33.70)
2	12 (30%)	48.05 (58.10, 32.80)
3	15 (37%)	53.40 (58.10, 45.60)
Iron grade (Perl’s iron stain)		
0	7 (17%)	59.10 (61.30, 56.60)
1	9 (23%)	56.50 (58.10, 46.40)
2	12 (30%)	53.96 (58.10, 46.70)
3	10 (25%)	46.20 (54.10, 34.40)
4	2 (5%)	(33.70, 32.80)
Fibrosis stage (**Masson’s trichome staining)**	6	
0	6 (15%)	59.35 (61.30, 51.10)
1	6 (15%)	55.80 (57.30, 46.10)
2	8 (20%)	53.45 (58.10, 46.40)
3	10 (25%)	52.10 (56.10, 46.10)
4	10 (25%)	42.20 (53.40, 32.80)

Note: Steatosis grade, iron grade, and fibrosis stage from liver biopsies of 40 SD rats, and the distribution of the T2 relaxation time of M-GRASE sequences in the pathology of the liver.

### Correlation and difference between the pathological grade of hepatic iron and T2 relaxation time

**Fig 4:**
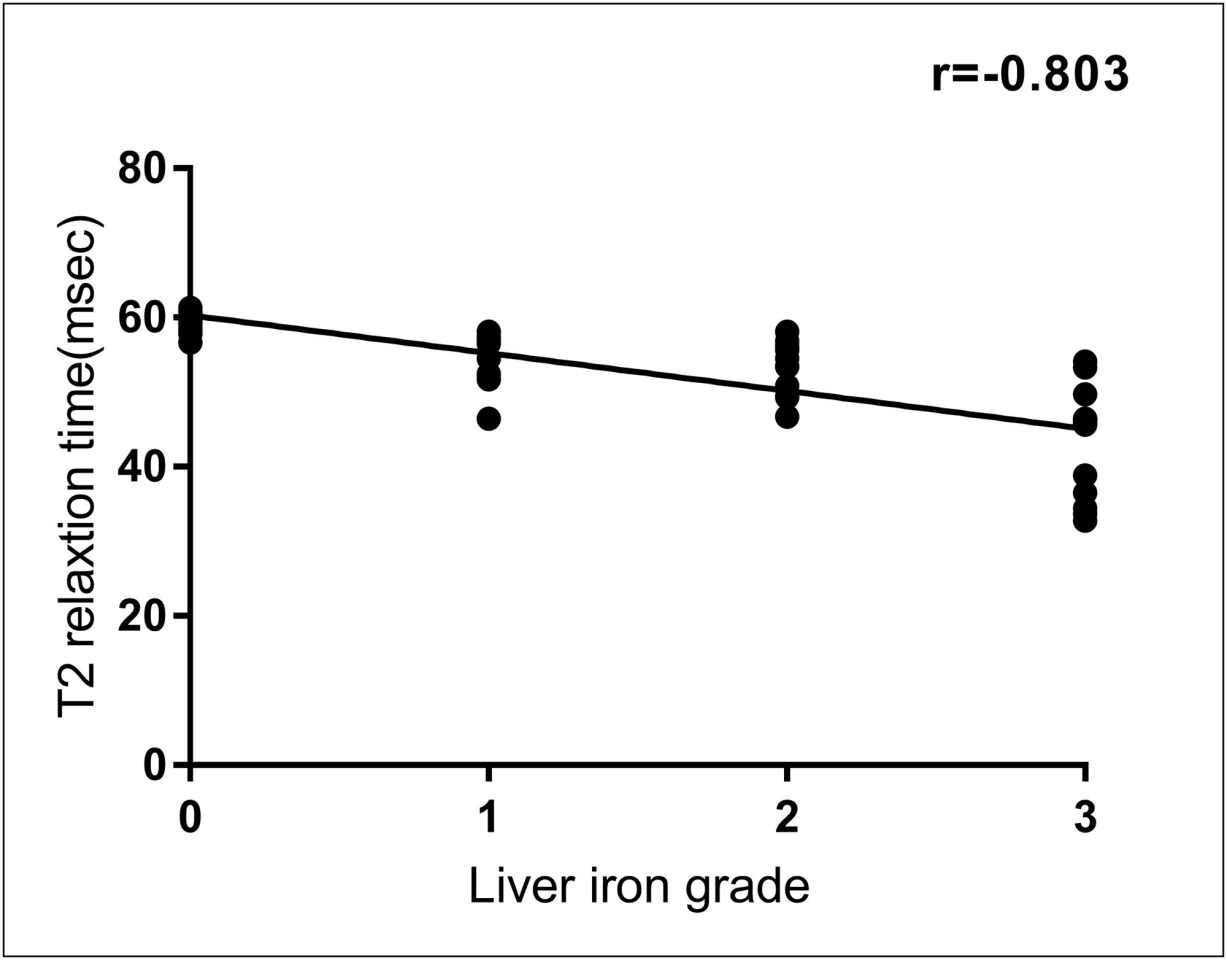
Correlation between T2 relaxation time and histologic iron grade. Scatterplot of T2 relaxation time versus subjective assessment of histologic iron grade reveals a strong correlation.

**Fig 5:**
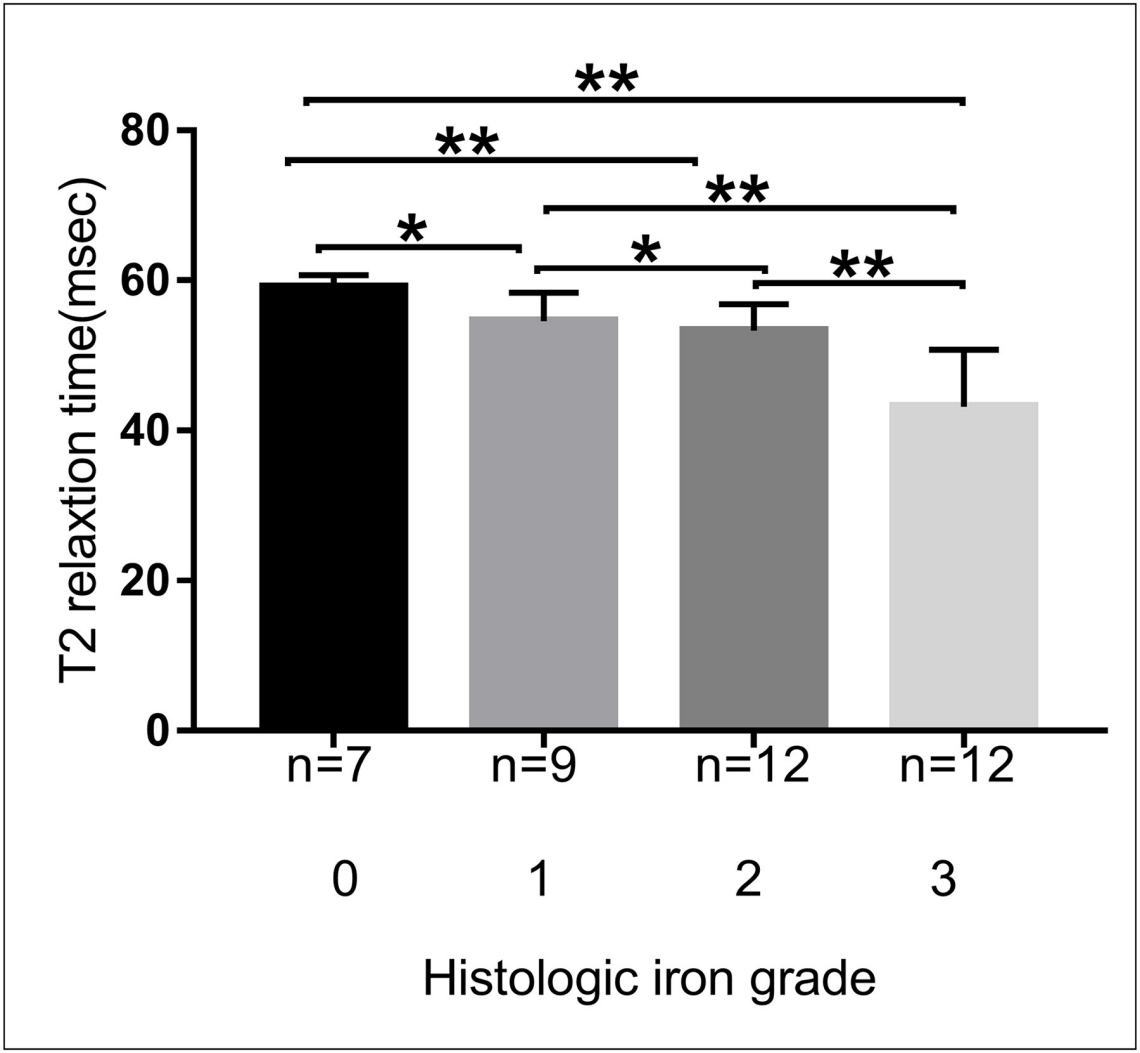
T2 relaxation time and histology. Bar graph representation: the horizontal bar represents the median, and the box represents the interquartile range. Based on the pairwise comparison between the liver T2 relaxation time and four histologically determined grades of liver iron, there were significant differences in T2 relaxation time between rats with different iron grades, except between grades 0 and 1 and between grades 1 and 2. *P < 0.*05* and **P > 0.*05*.

**Fig 6:**
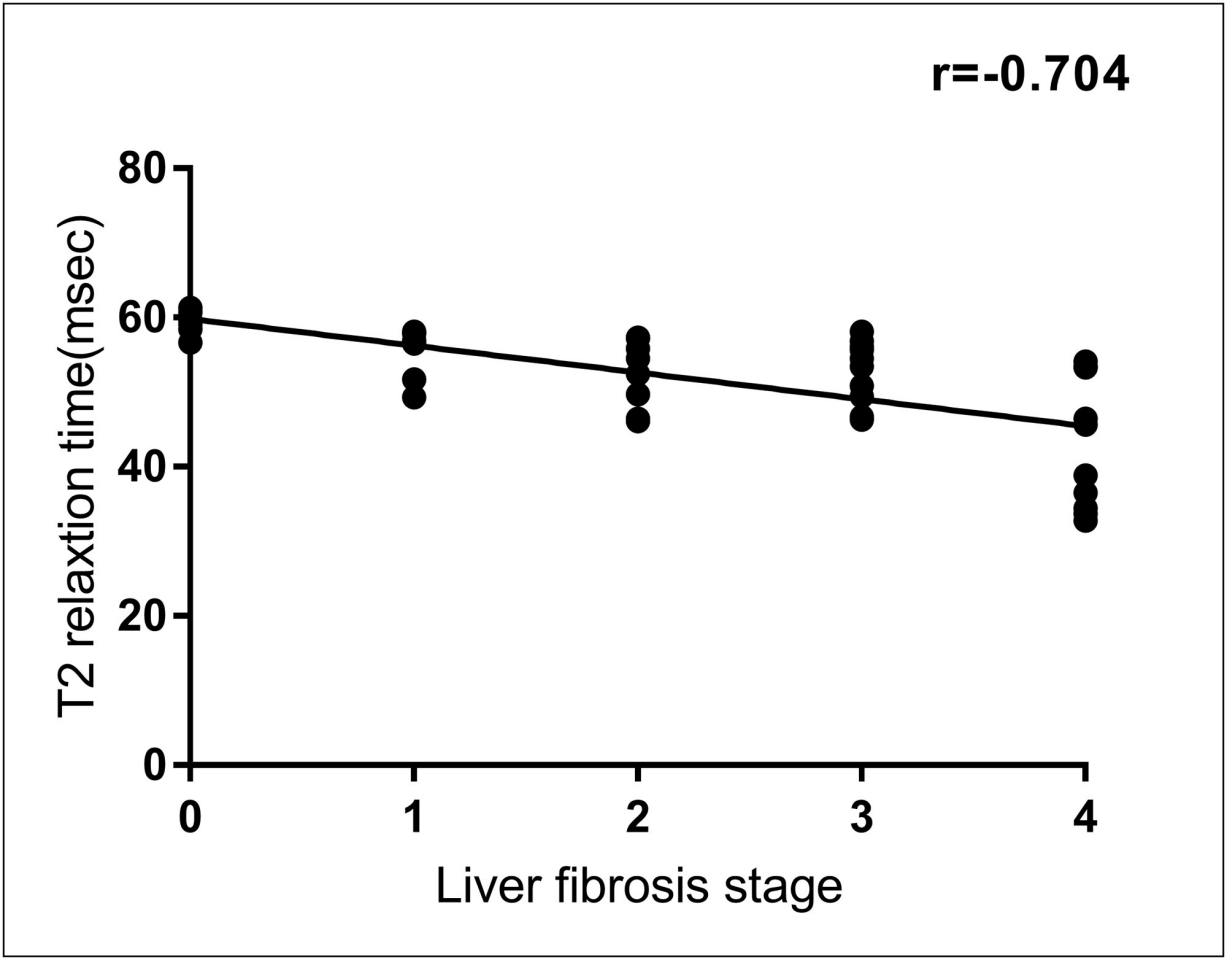
Correlation between T2 relaxation time and liver fibrosis stage. Scatter plot of T2 relaxation time versus subjective assessment of liver fibrosis stage reveals a moderately negative correlation.

**Fig 7:**
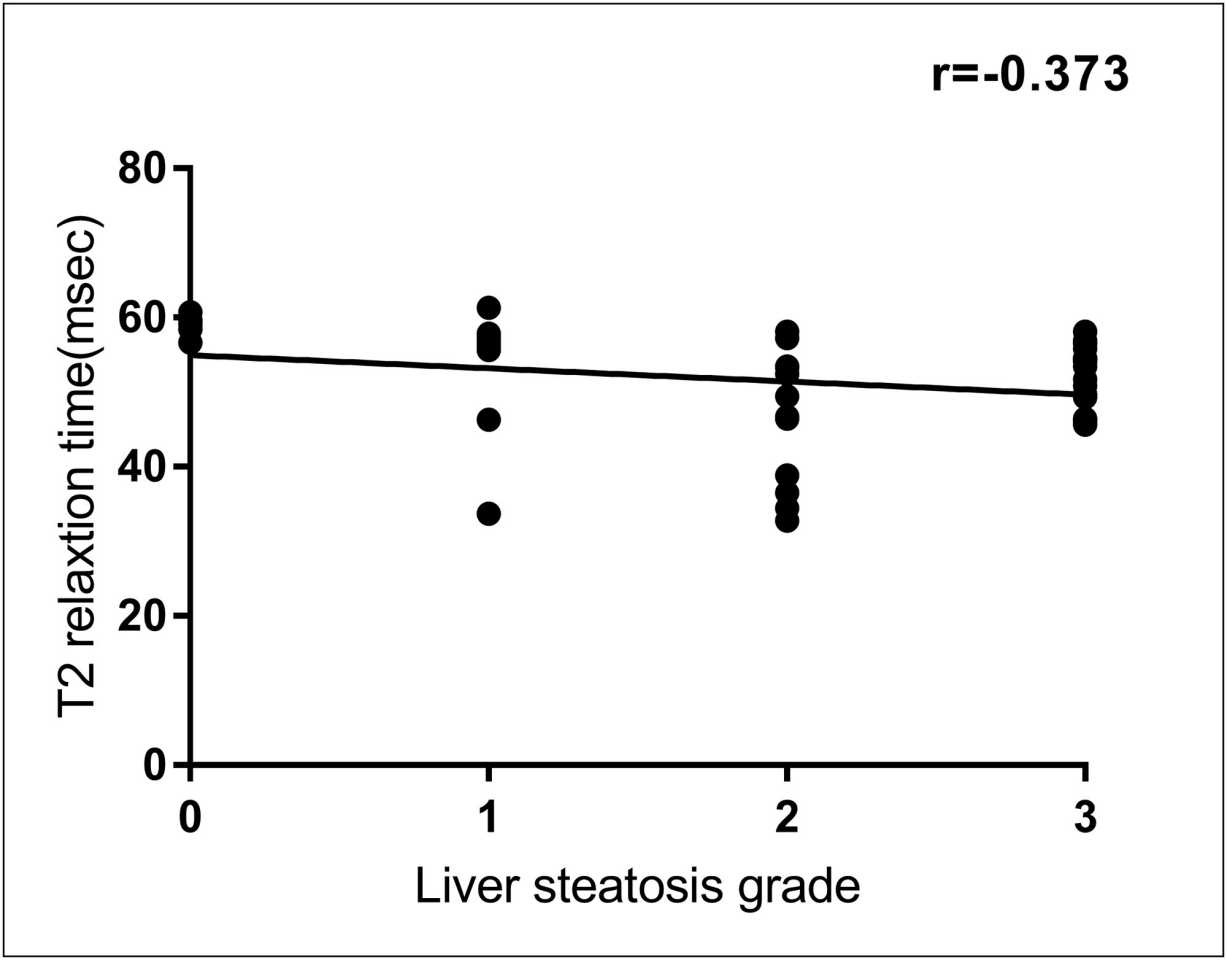
Correlation between T2 relaxation time and liver steatosis grade. Scatter plot of T2 relaxation time versus subjective assessment of liver steatosis grade reveals weak correlation.

A negative correlation was observed between iron pathological grade and T2 relaxation time (*r = −0*.*803*, *P < 0*.*001*), indicating that as the iron pathological degree increased, the T2 relaxation time gradually decreased (Fig 4). As demonstrated in Table 2, statistical assessment showed that there were highly significant differences in T2 relaxation time among rats with different histopathological grades of liver iron (*P < 0*.*001*; Mann–Whitney U). A Kruskal–Wallis test with Bonferroni correction (Fig 5) revealed significant differences in T2 relaxation time among rats with different iron grades (*P < 0*.*05* for iron grade 0 versus grade 2; *P < 0*.*05* for iron grade 0 versus grade 3; *P < 0*.*05* for iron grade 1 versus grade 3; and *P < 0*.*05* for iron grade 2 versus grade 3), except between rats with grades 0 and 1 and between rats with grades 1 and 2 (*P > 0*.*05*). Spearman rank correlation analyses (Figs 6 and 7) revealed that the T2 relaxation time was negatively correlated to the liver fibrosis stage (*r = −0*.*704*, *P = 0*.*000*); however, there was no correlation to the pathological grade of hepatic steatosis (*P > 0*.*05*). There were significant differences in T2 relaxation time among rats with different stages of liver fibrosis (*P < 0*.*05*). Figs 6 and 7 show the correlation between the T2 relaxation time and liver fibrosis stage and steatosis grade.

**Table 2:** The T2 relaxation times were compared among rats with different histologically determined iron grades based on a Mann–Whitney *U*-test.

Iron grade of liver	N (%)	T2 relaxation time (ms) Median (maximum, minimum)
0	7 (17%)	59.10 (61.30, 56.60)
1	9 (23%)	56.50 (58.10, 46.40)
2	12 (30%)	53.96 (58.10, 46.70)
≥3	12 (30%)	45.86 (51.10, 32.80)
*H*		26.367
*P*		0.001

Note: Significant at *P < 0*.*05*, the T2 relaxation times exhibited strongly significant differences among rats with different histologically determined iron grades.

### Diagnostic performance of T2 relaxation time in the pathological grading of hepatic iron

ROC analysis (Table 3) revealed a very high diagnostic value of the T2 relaxation time in the pathological grading of hepatic iron. The T2 relaxation time of liver had an AUC of 0.965 (95% CI 0.908–0.100, *P < 0*.*001*) for distinguishing rats with a pathological grade of hepatic iron (grade ≥ 1) from those without, an AUC of 0.871 (95% CI 0.757–0.985; *p < 0*.*001*) for distinguishing rats with no iron overload (grade ≤ 1) from rats with moderate or severe iron overload (grade ≥ 2), and an AUC of 0.939 (95% CI 0.865–1.000; *P < 0*.*001*) for distinguishing rats with no or mild to moderate iron overload (grade ≤ 2) from rats with severe iron overload (grade 3).

**Table 3:** Receiver operating characteristic (ROC) curves of T2 relaxation time for determining the pathological grade of hepatic iron.

	*0 vs*. *1–3*	*0–1 vs*. *2–3*	*0–2 vs*. *S3*
AUROC (95% CI)	0.965 (0.908,1.000)	0.871 (0.757,0.985)	0.939 (0.865,1.000)
Sensitivity (%)	100.00	75.00	96.43
Specificity (%)	81.82	91.67	75.00
Positive likelihood ratio (%)	5.50	9.00	3.86
Negative likelihood ratio (%)	0.00	0.27	0.05

### The main and interaction effects of liver steatosis, fibrosis, and iron on T2 relaxation time

According to Levene’s test, there was a homogeneity of variance (*P > 0*.*05*) between each group. The results of two-way analysis of variance for the interaction of fat, iron pathological grade, fibrosis stage, and T2 relaxation time were as follows: for the pathological grade of liver steatosis, *F = 0*.*627*, *P > 0*.*05*; for the pathological grade of hepatic iron, *F = 4*.*558*, *P < 0*.*05*; for the stage of liver fibrosis, *F = 0*.*359*, *P > 0*.*05*; and for their interactions, *F = 1*.*701*, *P > 0*.*05*. This suggested that the pathological grading of hepatic iron had a statistically significant effect on T2 relaxation time. In contrast, the pathological grading of hepatic fat and hepatic fibrosis staging did not have a statistically significant effect on T2 relaxation time, and the interaction of the pathological grading of hepatic fat and hepatic fibrosis staging did not have a statistically significant effect.

## Discussion

CCl_4_ is a toxic chemical that can cause hepatocyte acute and chronic injury, promote hepatic stellate cell (HSC) activation, reduce reticuloendothelial function, and cause changes in hematopoiesis and protein and fatty acid metabolism, leading to excessive accumulation of collagen and extracellular matrix, steatosis, and iron deposition [25, 26]. In our study, liver fibrosis was found to be related to iron accumulation in the liver, and iron grade was found to increase with fibrosis stage (*r = 0*.*810*, *P < 0*.*01*). There was no correlation between steatosis grade and fibrosis stage in the liver (*P > 0*.*05*). We also assessed the correlation between T2 relaxation time and fibrosis stage and showed that the T2 relaxation time was negatively correlated with the liver fibrosis stage (*r = −0*.*704*), which contradicted the findings of Guimaraes [27], who showed a monotonic increase in mean T2 value with increasing degree of fibrosis, possibly due to a lack of iron and fat deposition in their subjects. Wang [28] used the T2 value to assess the staging of CCl_4_-induced liver fibrosis in rats. The study found that the T2 value increased with the degree of fibrosis. However, this study did not assess hepatic iron deposition in rats, and regarding the rats this study, it is unclear whether iron deposition was present and, if present, its degree. The T2 values obtained in our study were different from those obtained in the previous studies, possibly due to inconsistencies in the equipment used for scanning, parameters, coils, and the basis of the lesions in the model. In the future, we can investigate how to optimize the selection of equipment, sequences, parameters, and coils in different pathological base models.

Alexopoulou et al. [19] used T2 relaxation time to study the iron concentration and iron pathological grade in iron solutions and high iron diet-induced iron overload model rats. They found that T2 liver relaxation time has a good linear correlation with liver iron content. A study on patients aged 1–18 years with normal and severe liver iron overload showed that R2 (R2 = 1/T2) is linearly correlated with the iron concentration in liver biopsy (*r = 0*.*87*, *P < 0*.*05*) and is reproducible, suggesting that R2 has good repeatability and accuracy in evaluating liver iron content [29]. Kritsaneepaiboon et al. used 1.5-T and 3.0-T MRI T2 values and T2* to compare 20 healthy controls and 42 patients with iron deposition and found that liver T2 values (3.0 T) and T2* (1.5 T) provide better evaluations of the liver iron grade and can be used as alternatives to needle biopsy [30]. In our study, a similar result was obtained: a significantly negative correlation was found between T2 relaxation time and the pathological grade of hepatic iron (*r = −0*.*803*, *P < 0*.*001*), indicating that iron overload resulted in a shorter T2 relaxation time than that for normal tissues. We conducted a pairwise comparative analysis of T2 relaxation time among rats with different pathological grades. The comparisons between the grades of liver iron and T2 relaxation time showed significant differences, except between grades 0 and 1 and between grades 1 and 2. The AUROC obtained by using the T2 relaxation time measurements was 0.965 for the prediction of hepatic iron deposits, and that for severe hepatic iron deposits (stage 3 or greater) was 0.939. The reasons for the lack of differences in T2 relaxation time between rats with grades 0 and 1 and between rats with grades 1 and 2 may be as follows: T2 relaxation time is an indirect measurement of hepatic iron overload. It reflects changes in the iron content of tissues because the relaxation time is affected by the concentration, distribution, and size of iron particles. However, liver fibrosis and fat deposits can also result in the T2 relaxation of tissues [27, 31], which will partially offset the effect of iron deposition. Hepatic iron overload in the liver and the secondary changes that manifest alongside progressive collagen deposition are important background changes in the development of liver fibrosis. Liver fibrosis firstly causes the recruitment and activation of inflammatory cells. During the process of liver fibrosis, intrahepatic fibrous septal hyperplasia then creates the aggravation of hepatic inflammatory necrosis. Liver inflammation and fibrosis can limit the diffusion and movement of water molecules and reduce the sensitivity of T2 relaxation time to the diagnosis of iron overload. In our study, rats with early liver fibrosis induced by CCl_4_ had fewer iron deposits and less uneven and severe fat deposits, which may have affected the accuracy of the T2 relaxation time identification of iron grade to a certain extent. As fibrosis progressed, liver cell edema and steatosis decreased, and hepatic iron deposits increased. With the significant retention of iron in hepatocytes at grades 3 and 4, the T2 relaxation time of the liver was significantly shortened. Patients with liver iron deposition are often associated with fibrosis and the presence of fat. In recent years, researchers have begun to study the effects of fat and fibrosis on iron quantification. França et al. used 3.0-T MECSE-MR to simultaneously quantify the liver steatosis and siderosis of 101 patients with diffuse liver disease. They found that liver R2^*^ measurement has a significant positive correlation with the pathological grade of iron (R2^*^ = 1/T2^*^). The R2^*^ value quantifies liver siderosis and is not affected by liver fibrosis [32]. Similarly, Papakonstantinou et al. concluded that liver T2 values were more accurate than serum ferritin in predicting liver iron overload and were not influenced by the presence of chronic hepatitis C [20]. Another study also found that the stage of liver fibrosis and grade of necroinflammation did not have a significant effect on SDPA R2-MRI and biopsy-measured LIC [18]. In our experiments, according to a significance level of 5% (α = 0.05), the interaction of iron pathological grade, fibrosis stage, and steatosis was not significant (*F = 1*.*710*, *P > 0*.*05)*, indicating that in the presence of fibrosis, fat, and iron, the T2 relaxation time is mainly affected by iron. However, some research findings differ from ours. Li et al. [33] established in vitro water phantoms with various concentrations of gadolinium (Gd), collagen (Cl; modeling fibrosis), and fat to investigate the interfering effects of fibrosis and fat on the R2^*^ estimation of LIC by comparing R2^*^ with QSM. The study revealed that the R2* estimation of LIC is prone to substantial nonlinear interference from fat, fibrosis, and other lesions. This differs from our findings, possibly because the research by Li et al. is based on the study of the interference effect between a fixed iron concentration of [Gd] = 1.25 mmol/L and increasing collagen and fat concentrations, whereas in the in vivo model we studied, the iron content was not fixed but distributed, with no obvious positive correlation between iron and fat.

Our study has the following shortcomings. First, the sample size in this study was relatively small, and the diagnostic threshold of the T2 relaxation time for the pathological grading of iron was not obtained. Second, due to the limitation of conditions, the iron and pathological grading of rat liver were semi-quantitative indicators for visual analysis. Spectrophotometry and Soxhlet chemistry extraction methods can provide a more accurate quantitative detection of liver iron and fat. Finally, and most importantly, our findings suggest that the coexistence of iron, fibrosis, and fat in the in vivo model is another limitation of our study. However, we did not conduct studies on the effect of only-iron, only-fibrosis, and only-fat on T2 relaxation time. In the future, we will increase the sample size and use spectrophotometry to quantify liver iron content to further determine the best diagnostic threshold of different grades of liver iron deposition levels using T2 relaxation time. We will also study only-iron, only-fibrosis, and only-fat samples to obtain more conclusive results.

## Conclusion

In our study, which included 40 SD rats with hepatic steatosis, fibrosis, and iron overload, as the liver fibrosis stage progressed, the liver iron grade increased. Lower T2 relaxation time in rats with liver fibrosis may be correlated with a higher pathological iron grade, which may be an alternative method to non-invasive MRI for assessing the iron grade in liver fibrosis. T2 relaxation time can distinguish liver iron grades in the presence of liver steatosis or fibrosis. Furthermore, T2 relaxation time is suitable for distinguishing between the presence and absence of iron deposition. It can also provide a more accurate distinction of higher iron grades.

## Supporting information

S1 File (ZIP)
