# Supplementary material for: Diagnostic value of T2 relaxation time for hepatic iron grading in rat model of fatty and fibrotic liver
Source: PLoS One. 2022 Dec 5;17(12):e0278574. doi: 10.1371/journal.pone.0278574 (PMC9721484; doi:10.1371/journal.pone.0278574)
Supplement: S1 File — (ZIP) [file pone.0278574.s001.zip › Supporting informaion/Laboratory Protocols .docx]

**Materials and Methods**

**Carbon Tetrachloride Edible Blended Oil Suspension (Solution)**

CCl_4_ solution (99.5% concentration, 80.4 ml) was diluted in edible blended oil (200 ml) in a brown bottle to obtain a suspension concentration of 40%. CCl_4_ was purchased from Jiangsu Qiangsheng Functional Chemical Co, Ltd. (Because high-purity CCl_4_ solution has strong volatility and toxicity, experimenters wore plastic gloves and disposable masks throughout the process and kept the laboratory ventilated.) The suspension was placed in a cool, dry environment away from light before MRI measurement.

The laboratory animal production licenses were SCXK (Chuan) 2013-17, SCXK (Chuan) 2013-181, and SCXK (Chuan) 2013-065. Sixty male Sprague-Dawley (SD) rats (2–3 months old), weighing 202 ± 3.2 g, were provided by the Experimental Animal Centre of Southwest Medical University (Sichuan, China). The experimental procedures mentioned in this study were approved by the Ethical Committee for the Experimental Use of Animals at Southwest Medical University (Sichuan, China). All rats were fed a normal diet and housed under controlled conditions. Rats were injected with the 40% CCl_4_ edible mixed oil suspension (0.3 ml/100 g) subcutaneously into the abdominal cavity or the inside of the hind legs twice a week. From 6 to 12 weeks of modeling, 1–6 rats with light weight, darker coat color, and listlessness were selected for MRI scan every week. For MRI, the rats were anesthetized by subcutaneous injection of 2% sodium pentobarbital (0.2 ml/100 g).

MRI was performed with an 8-channel wrist coil on a 3.0 Tesla MRI scanner (Achieva 3.0T, Philips Healthcare, The Netherlands). The pulse sequence was M-GRASE, and the parameters were: TE = 17 ms × 4 echos (TE1 = 17, TE2 = 34, TE3 = 51, and TE4 = 68 ms), TR = 1552 ms, slice thickness = 2.5 mm, slice gap = 1 mm, matrix size = 240 × 240, field of view (FOV) = 100 × 60 mm, number of excitations (NEX) = 1, and the scanning ranged from the top of the diaphragm to the lower edge of the kidneys. The rats were sacrificed via subcutaneous injection of 4% sodium pentobarbital (0.2 mL/100 g) after scanning.

**Histopathological Assessment**

The rats were killed immediately after MRI, and liver specimens were extracted, routinely processed with normal saline solution flushing and 10% buffered neutral formalin fixation and embedded in paraffin. Sections of the liver tissue were stained with hematoxylin and eosin (H&E) for histological evaluation of steatosis, Perl’s iron stain (Prussian blue reaction) for evaluation of iron grade, and Masson’s trichrome stain for evaluation of fibrosis. The outcomes of H&E, Prussian blue, and Masson’s trichome staining were independently evaluated by two experienced pathologists who were blinded to each other’s findings and MRI data. The histopathological outcome of each specimen represents the consensus opinion.

Diagnostic criteria for pathological grading of hepatic steatosis ^[23]^ were as follows: hepatic fat accumulation was evaluated using low-to-medium-power magnification estimation of hepatocyte involvement by macrosteatosis or microsteatosis with grade 0 at <5% of parenchyma, grade 1 at 5%–33%, grade 2 at 34%–66%, and grade 3 at >66%. Hepatocellular iron was graded as follows ^[24]^: grade 0 if there are no iron particles; grade 1 if iron particles can be seen in a few hepatocytes; grade 2 if iron particles can be seen in 5%–10% of hepatocytes; grade 3 if >40% iron particles can be seen in hepatocytes; and grade 4 if abundant iron particles can be seen in most hepatocytes. Liver fibrosis was assessed by Scheuer Classification ^[25]^: F0 means no fibrosis; F1 means fibrous expansion of portal areas without short fibrous septa; F2 means fibrous expansion of most portal areas with occasional portal to portal bridging; F3 means marked bridging with occasional nodules and incomplete cirrhosis; and F4 means cirrhosis.

**Quantitative Image Analysis**

Raw image data were manually transferred to a PC workstation (Philips Extended MR WorkSpace, release 2.6.3.4; Philips Medical Systems), and the system automatically constructed a T2 map. Because of the inferiority in soft tissue contrast of T2 mapping, a circular region of interest (ROI) of 10 mm^2^ was placed on the T2 MRI (TE: 17 ms) first, and then the ROI of the same area was copied to the T2 map. The ROI was selected to be located in the larger cross-section of the liver and at the center of the images, and it was also selected to avoid artifacts, bile ducts, large blood vessels, and focal liver damage to reduce error. The ROI was set at three different locations of the same cross-section image of the T2 map. The T2 relaxation times of the MR images were independently measured by the two experienced radiologists, who were blinded to each other’s MR assessments and the results of the histological examination. The mean of the measurements was taken as the final value.

**The position of a region of interest (ROI) on the T2 map of liver.** Representative axial multi-echo gradient and spin echo (TE = 17 ms) T2 MRI imaging (a) and T2 map (b) of one SD rat illustrating the position of ROIs in the liver. Each ROI is located at the center of the largest cross-section of the liver and avoids artifacts, bile ducts, large blood vessels, and focal liver damage. Each ROI is first placed on the TE = 17 ms image and then copied to the same area of the T2 map. Three ROIs are placed in different positions on the same axial image.
