# Supplementary figures and images for: Diagnostic value of T2 relaxation time for hepatic iron grading in rat model of fatty and fibrotic liver
Source: PLoS One. 2022 Dec 5;17(12):e0278574. doi: 10.1371/journal.pone.0278574 (PMC9721484; doi:10.1371/journal.pone.0278574)

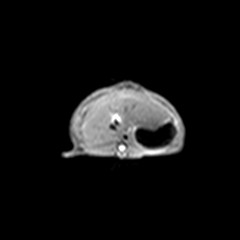

Supplement: S1 File — (ZIP) [file pone.0278574.s001.zip › Supporting informaion/Figure 1A in File S1.jpg]

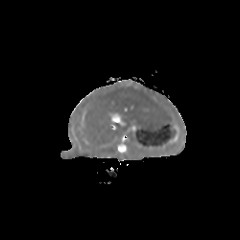

Supplement: S1 File — (ZIP) [file pone.0278574.s001.zip › Supporting informaion/Figure 1B in File S1.jpg]

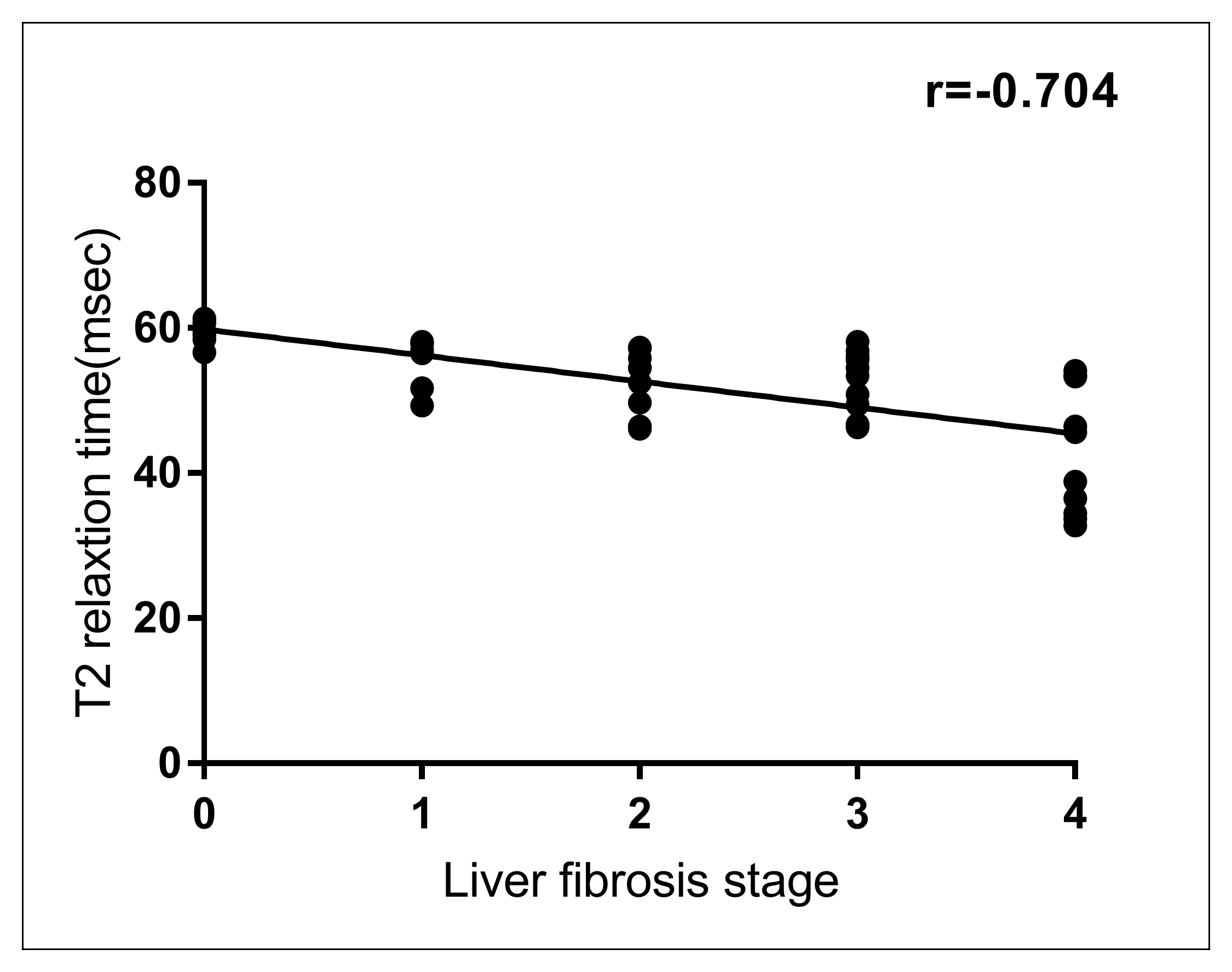

Supplement: S1 File — (ZIP) [file pone.0278574.s001.zip › Supporting informaion/Figure 6 in File S1.tif]

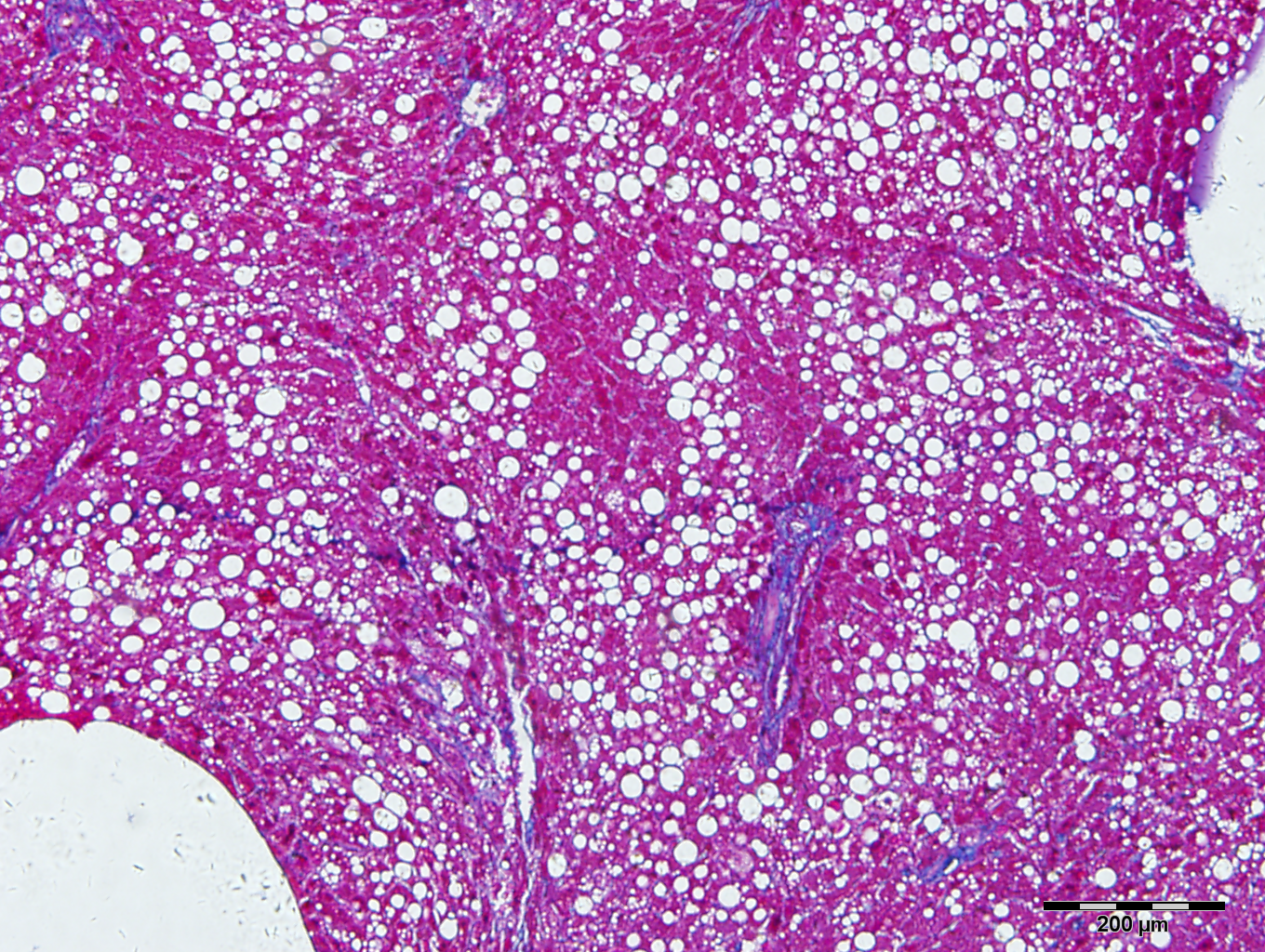

Supplement: S1 File — (ZIP) [file pone.0278574.s001.zip › Supporting informaion/Figure 2B in File S1.tif]

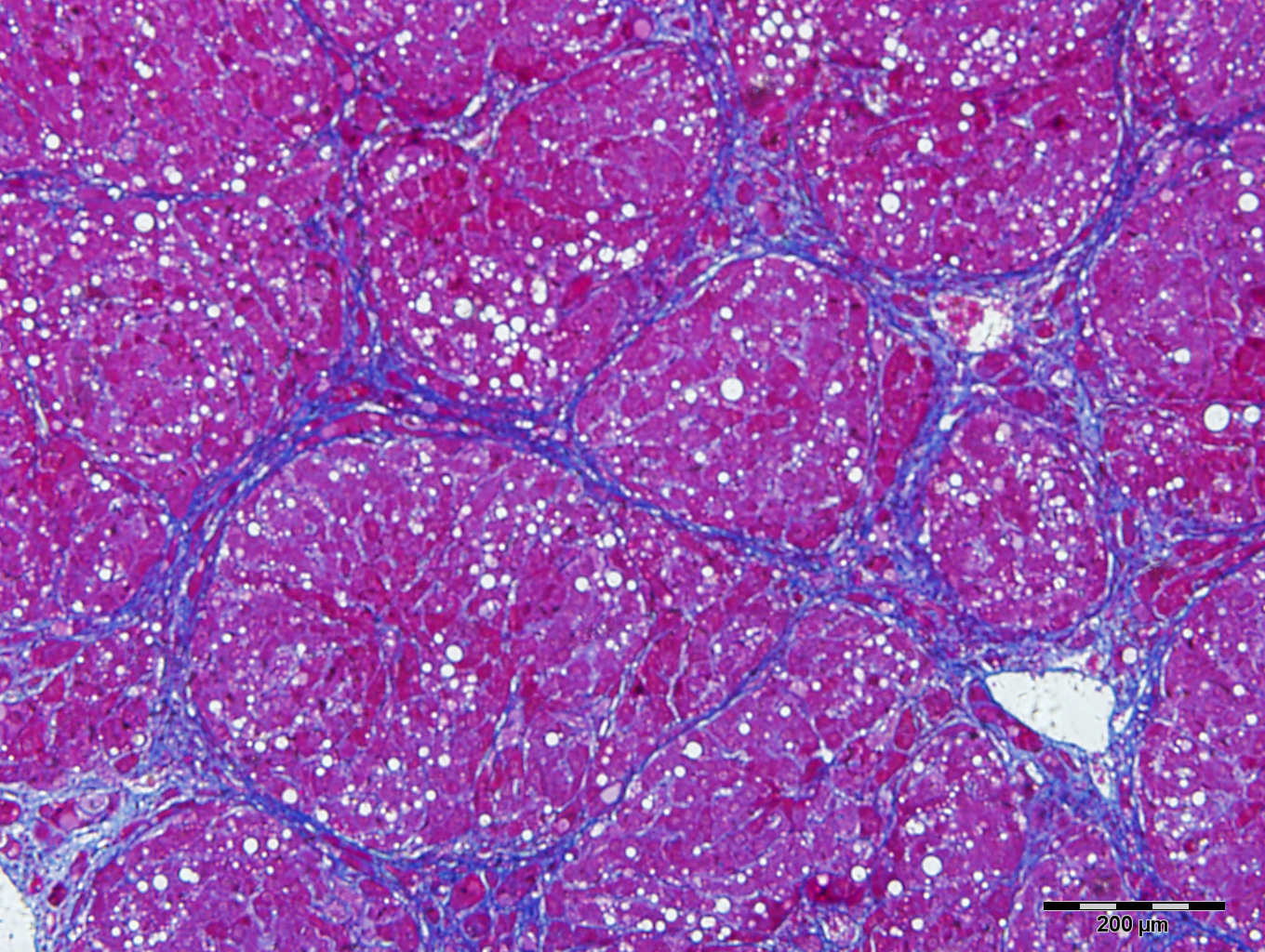

Supplement: S1 File — (ZIP) [file pone.0278574.s001.zip › Supporting informaion/Figure 2D in Flie S1.tif]

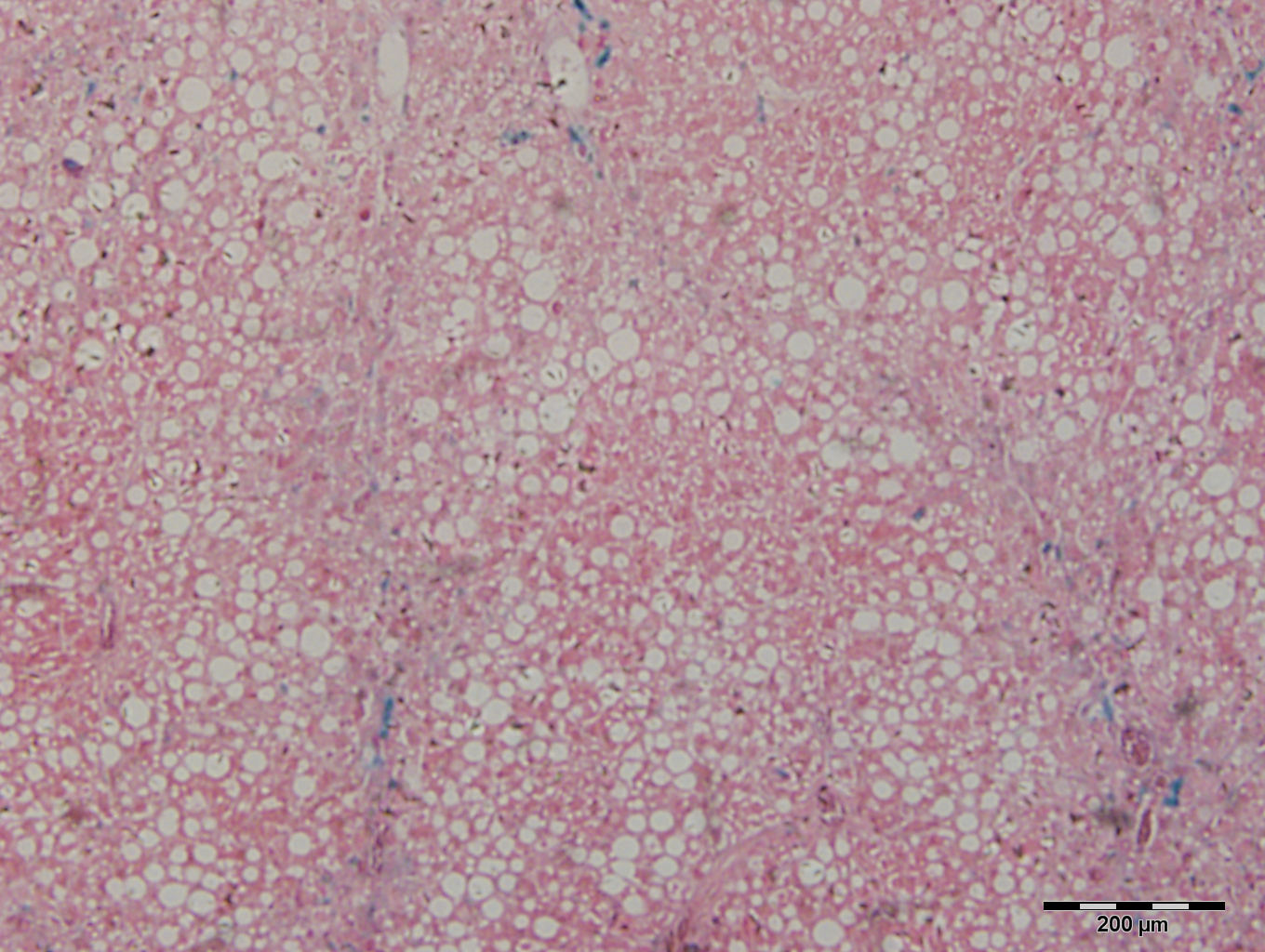

Supplement: S1 File — (ZIP) [file pone.0278574.s001.zip › Supporting informaion/Figure 3A in Flie S1.tif]

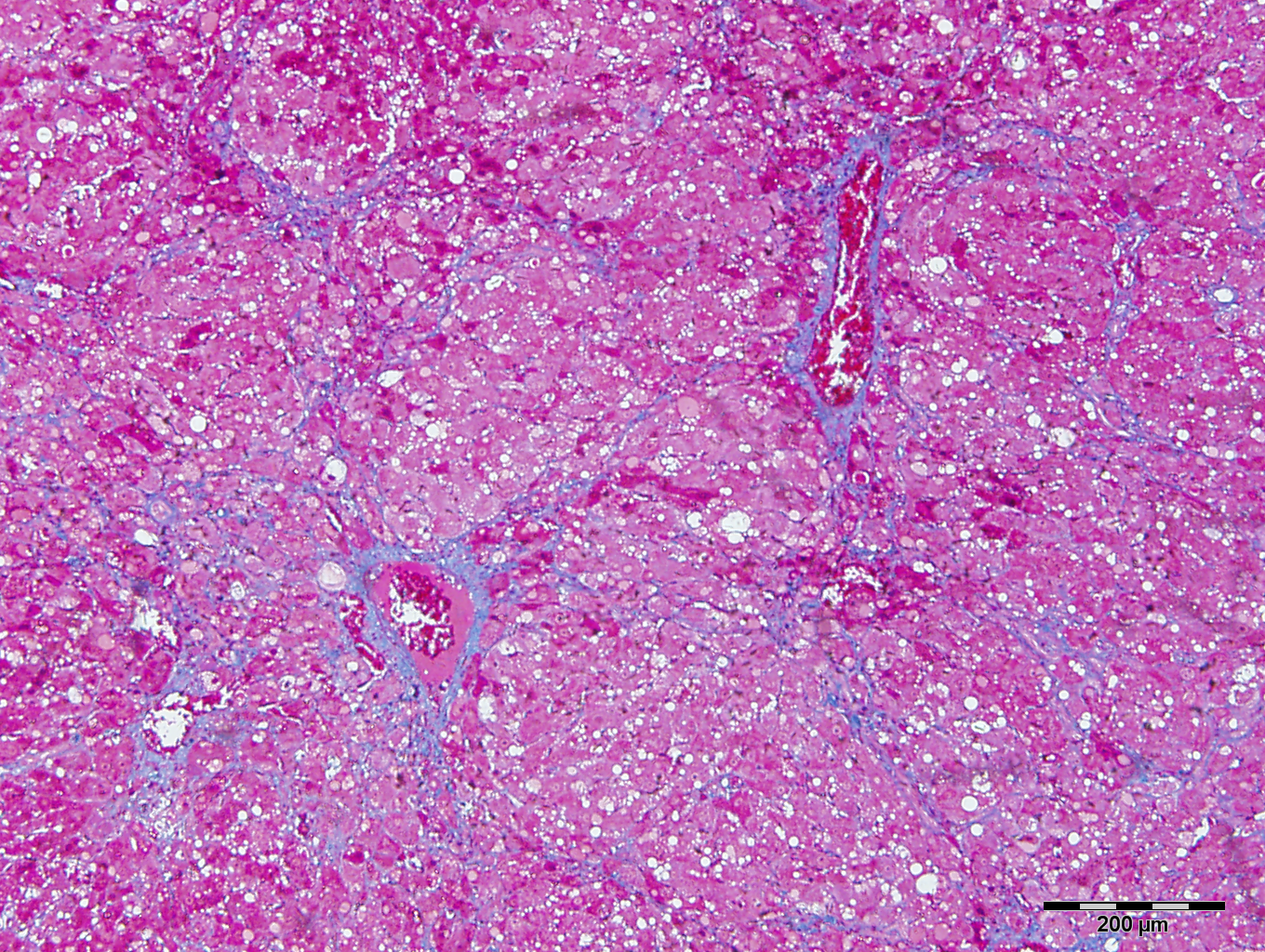

Supplement: S1 File — (ZIP) [file pone.0278574.s001.zip › Supporting informaion/Figure 2C in File S1.tif]

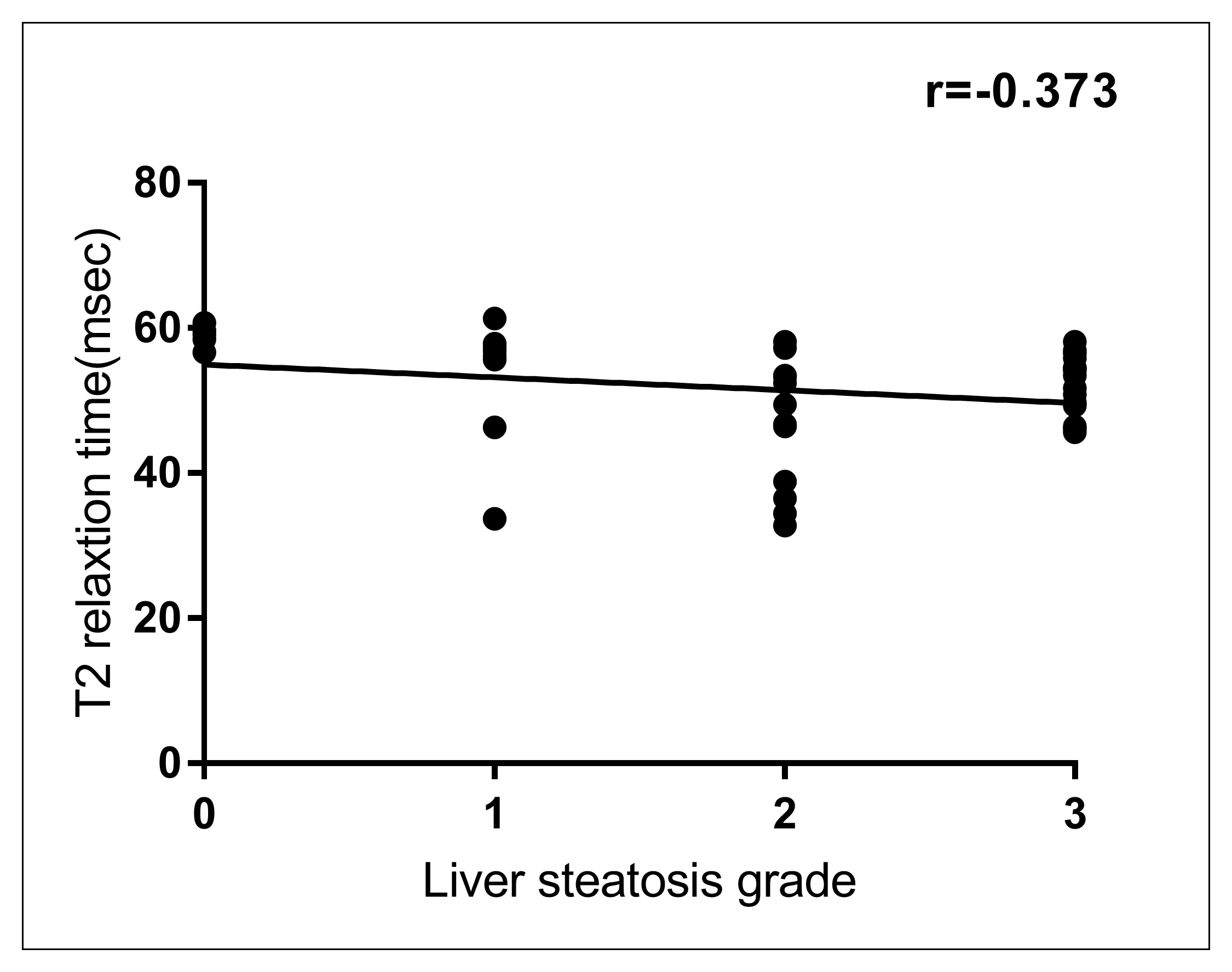

Supplement: S1 File — (ZIP) [file pone.0278574.s001.zip › Supporting informaion/Figure 7 in File S1.tif]

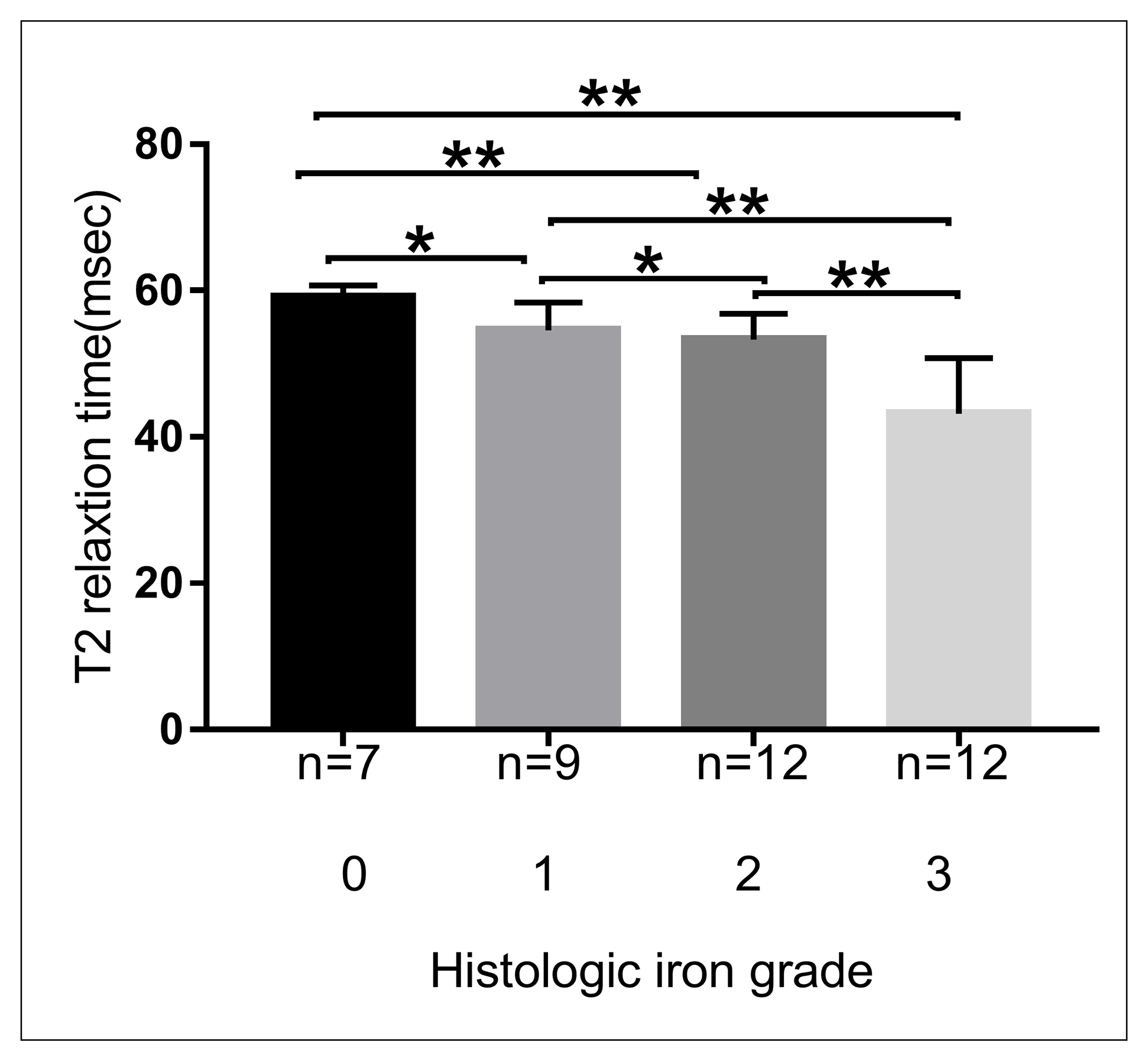

Supplement: S1 File — (ZIP) [file pone.0278574.s001.zip › Supporting informaion/Figure 5 in File S1.tif]

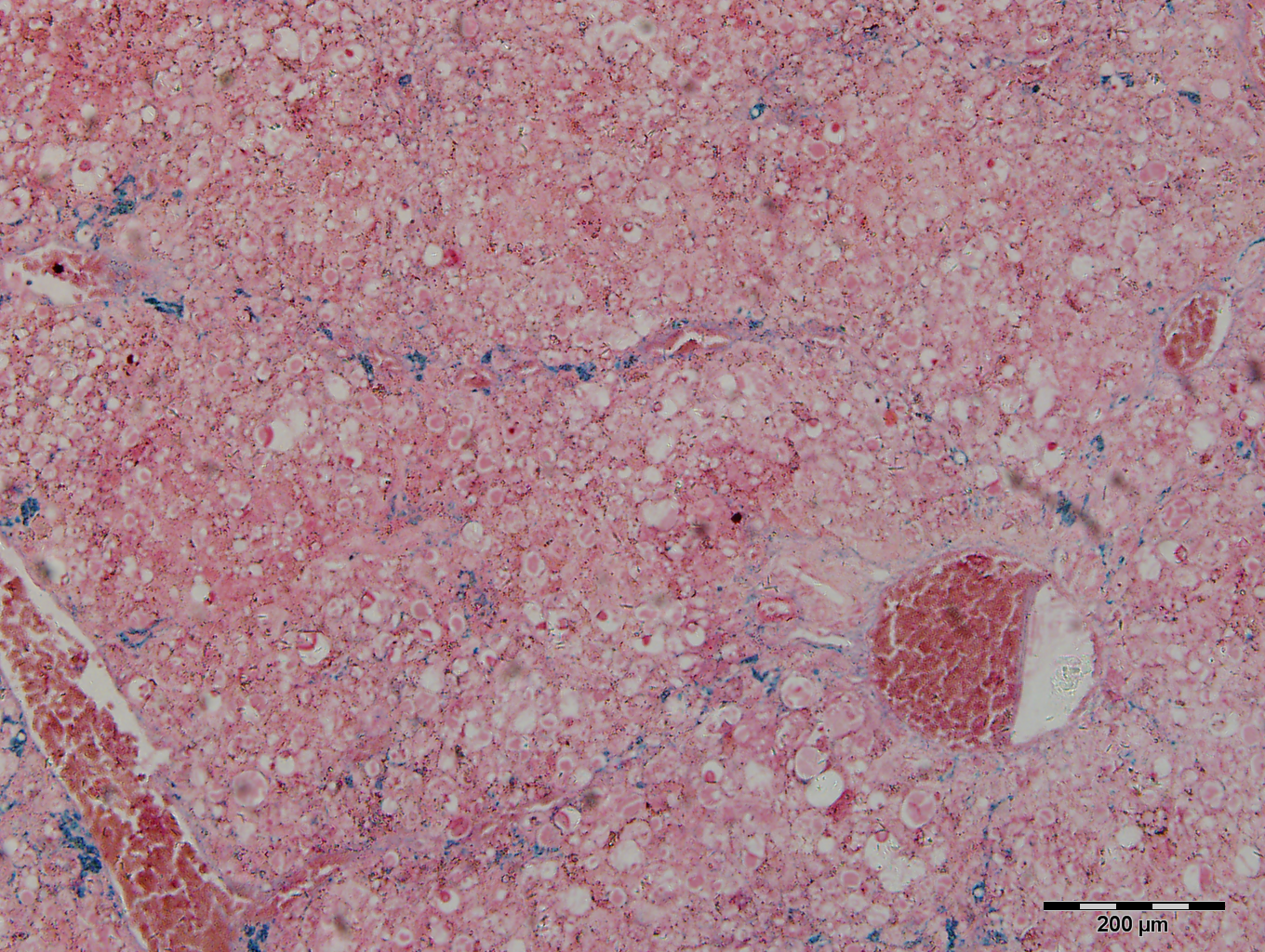

Supplement: S1 File — (ZIP) [file pone.0278574.s001.zip › Supporting informaion/Figure 3C in File S1.tif]

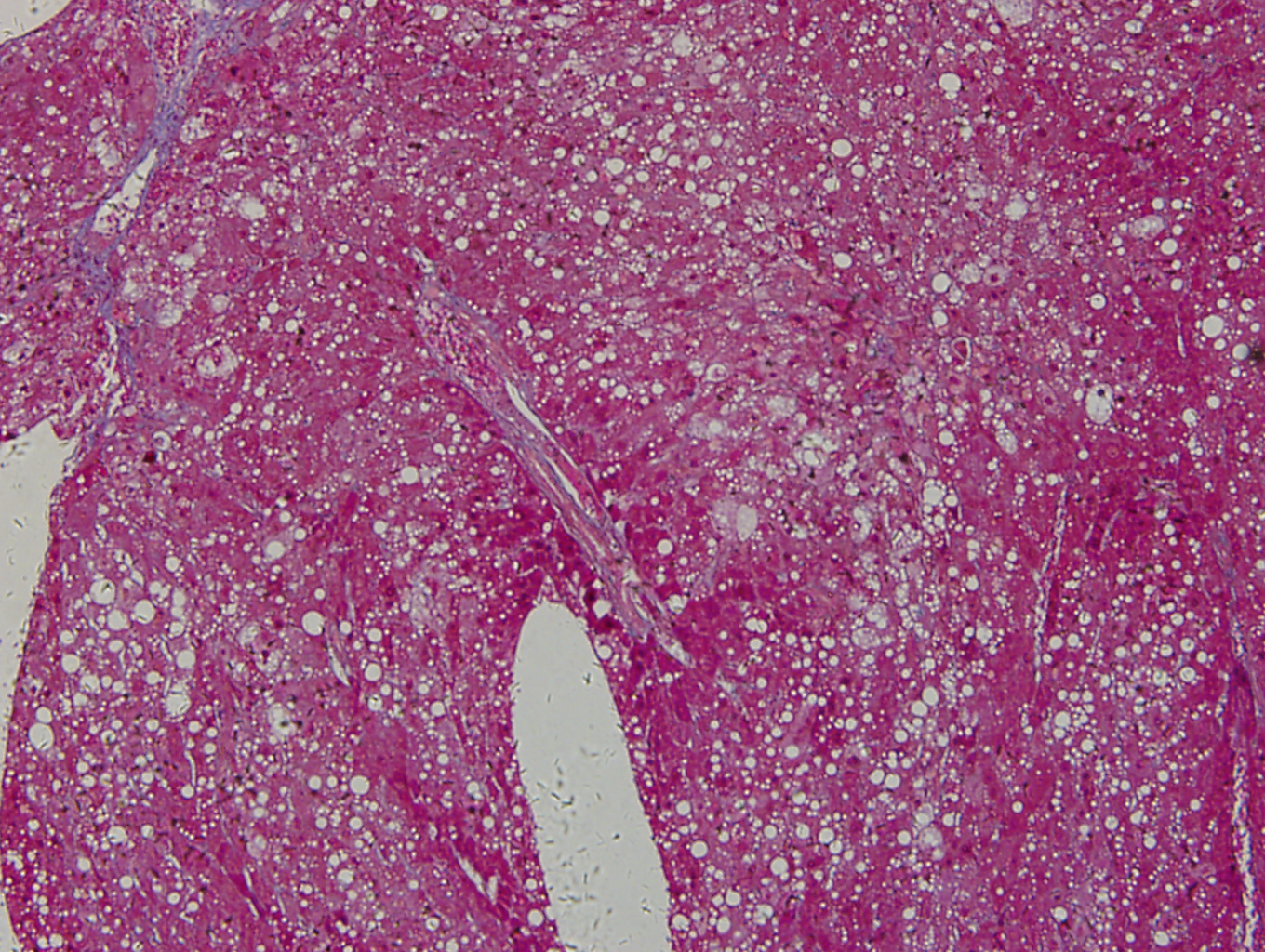

Supplement: S1 File — (ZIP) [file pone.0278574.s001.zip › Supporting informaion/Figure 2A in File S1.tif]

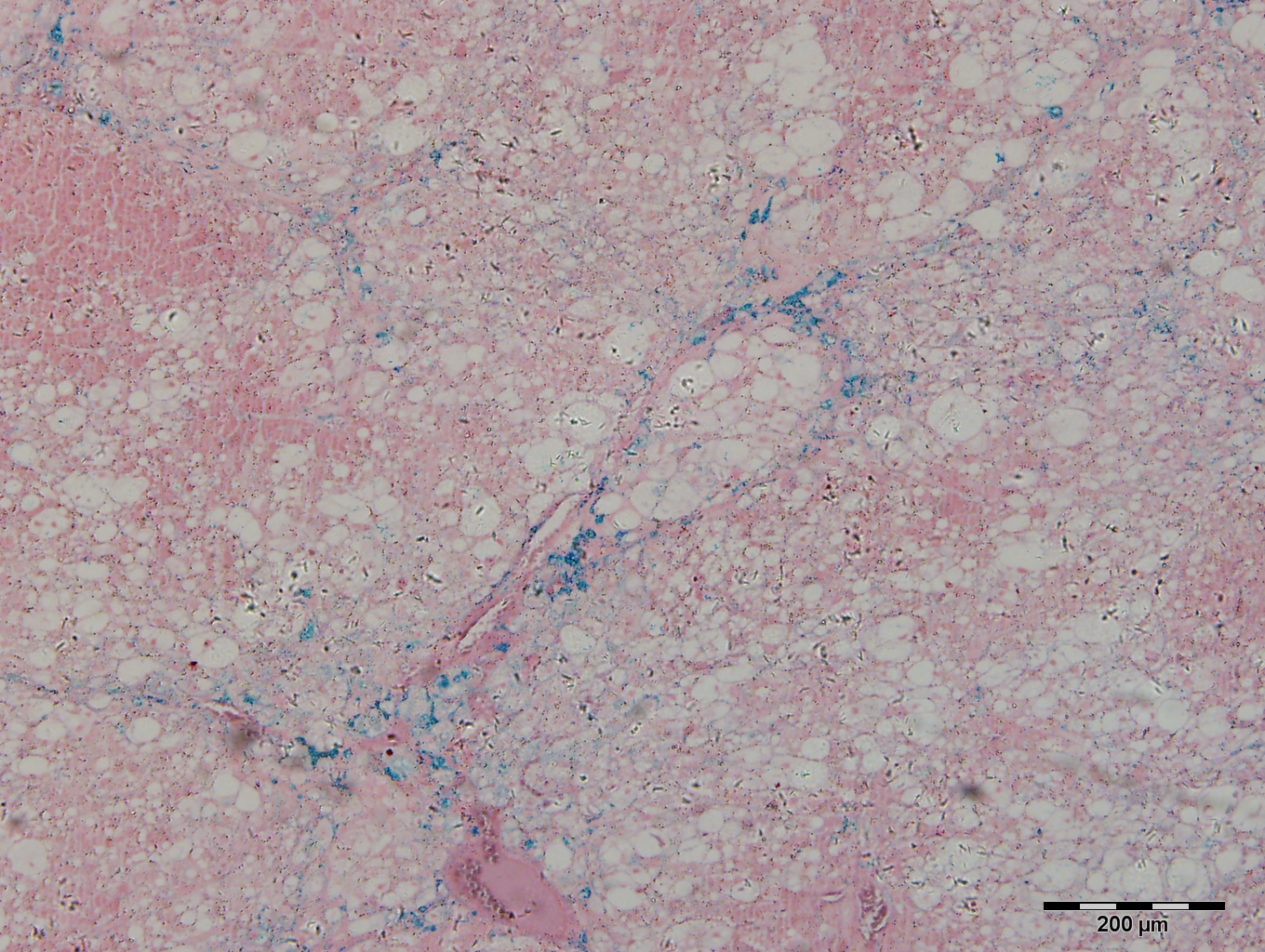

Supplement: S1 File — (ZIP) [file pone.0278574.s001.zip › Supporting informaion/Figure 3D in File S1.tif]

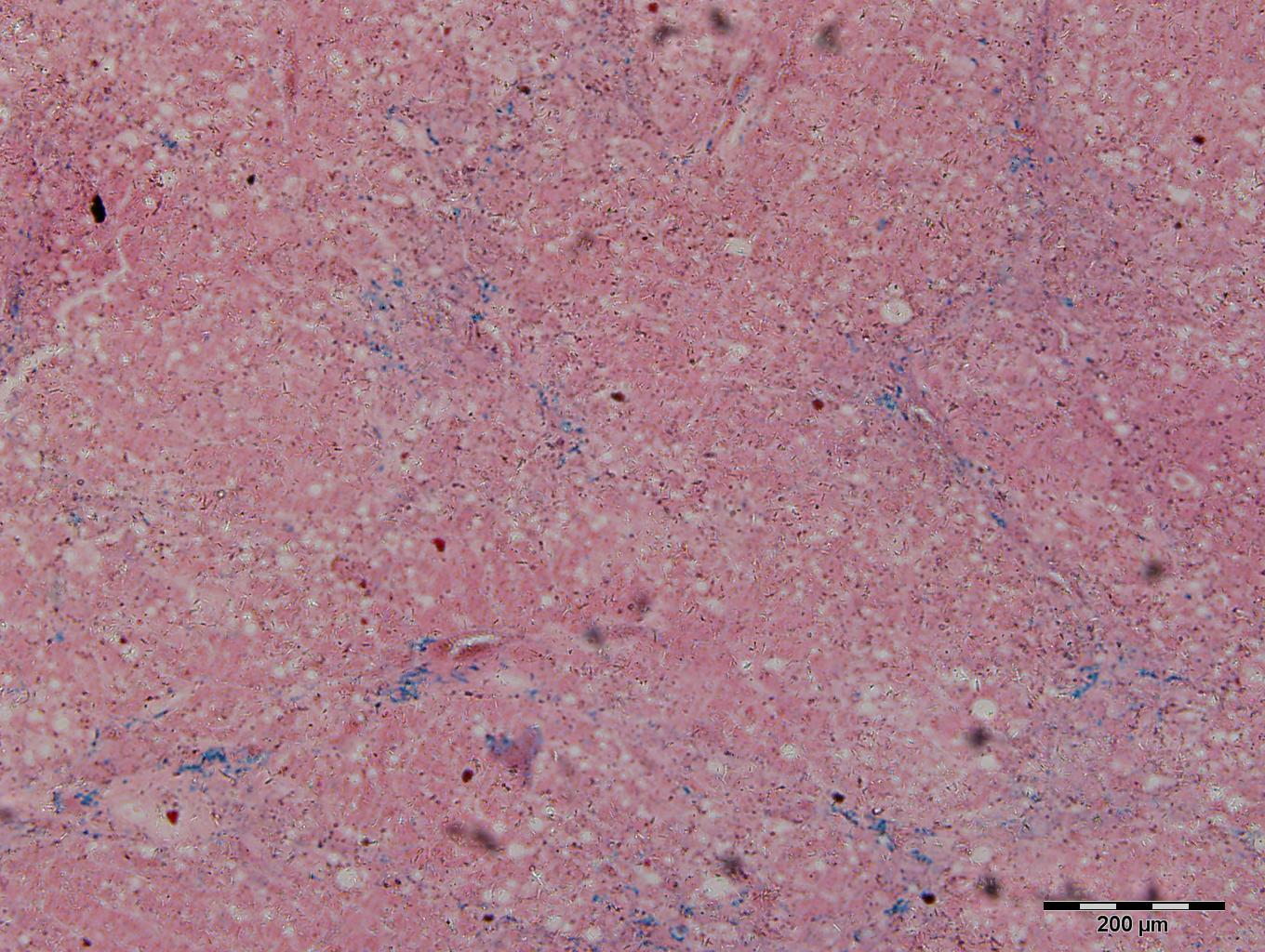

Supplement: S1 File — (ZIP) [file pone.0278574.s001.zip › Supporting informaion/Figure 3B in Flie S1.tif]

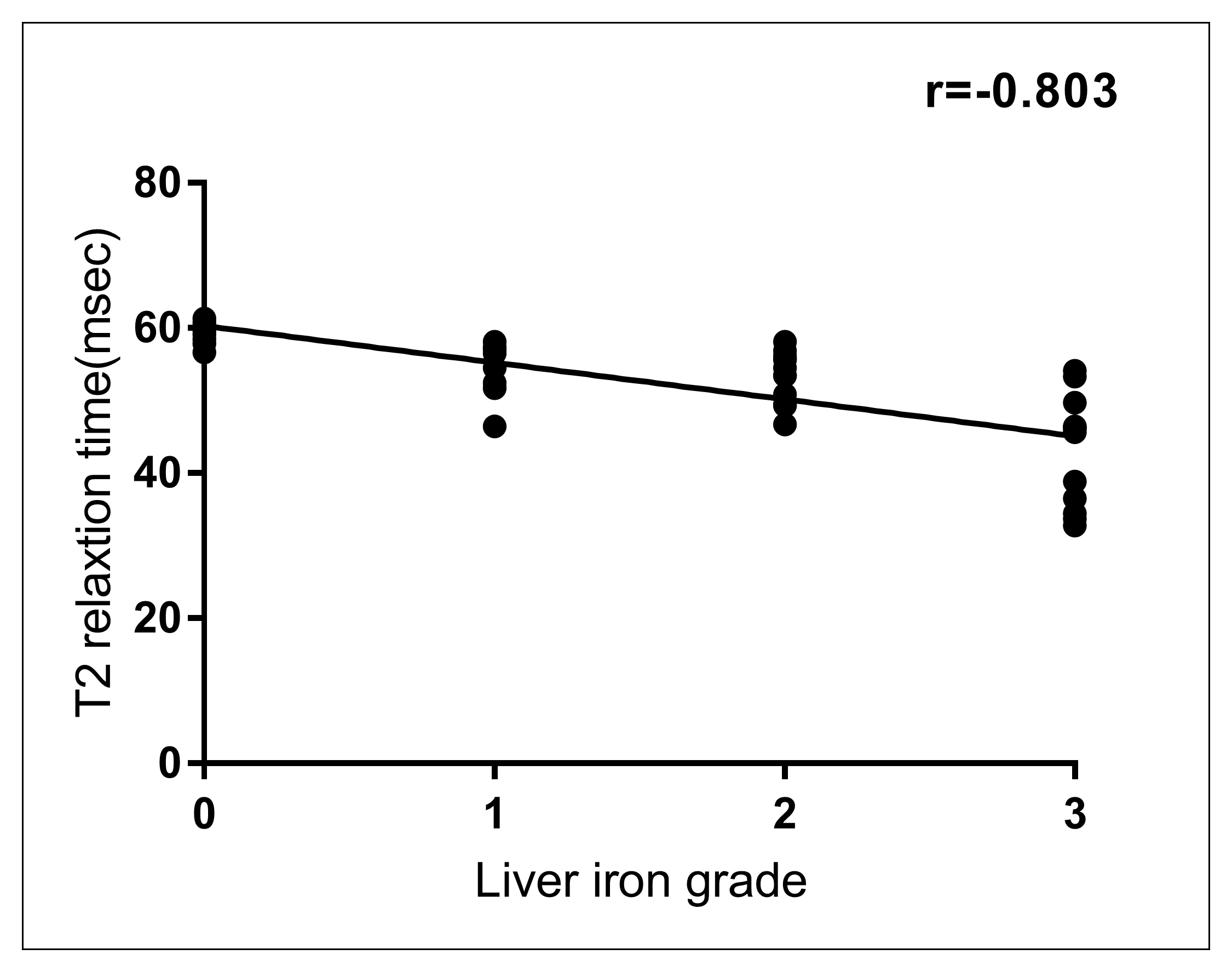

Supplement: S1 File — (ZIP) [file pone.0278574.s001.zip › Supporting informaion/Figure 4 in File S1.tif]
